# Magnetic Non-invasive Auricular Acupuncture During Eye-Exam for Retinopathy of Prematurity in Preterm Infants: A Multicentre Randomized Controlled Trial

**DOI:** 10.3389/fped.2020.615008

**Published:** 2020-12-23

**Authors:** Kimberly M. L. Gan, Ju-Lee Oei, Im Quah-Smith, Azanna A. Kamar, Alexis A. D. Lordudass, Kian D. Liem, Kwee Bee Lindrea, Mary Daly, Nilima Gaunker, Avneet K. Mangat, Maryna Yaskina, Georg M. Schmölzer

**Affiliations:** ^1^Faculty of Medicine, University of New South Wales, Kensington, NSW, Australia; ^2^School of Women's and Children's Health, University of New South Wales, Kensington, NSW, Australia; ^3^Department of Newborn Care, The Royal Hospital for Women, Randwick, NSW, Australia; ^4^Roseville Wellness Group, Roseville, NSW, Australia; ^5^Neonatology Unit, University of Malaya Medical Centre, Kuala Lumpur, Malaysia; ^6^Department of Neonatology, Radboudumc Amalia Children's Hospital, Nijmegen, Netherlands; ^7^Neonatal Research Unit, Centre for the Studies of Asphyxia and Resuscitation, Royal Alexandra Hospital, Edmonton, AB, Canada; ^8^Department of Paediatrics, University of Alberta, Edmonton, AB, Canada; ^9^Women and Children's Health Research Institute (WCHRI), University of Alberta, Edmonton, AB, Canada

**Keywords:** newborn, retinopathy of prematurity, pain, acupuncture, acupunctural analgesia

## Abstract

**Background:** Eye exam for Retinopathy of prematurity (ROP) is a painful procedure and pharmacological analgesia might be ineffective. We hypothesized that magnetic auricular acupuncture (MAA) compared to placebo will decrease pain during ROP exam in preterm infants.

**Methods:** Multicentre randomized controlled trial conducted in three hospitals (Australia, Canada, and Malaysia). Eligibility: >32 weeks, ROP exam, not sedated, and parental consent. A total of 100 infants were randomized (1:1) to MAA (*n* = 50) or placebo (*n* = 50). MAA stickers or placebo were placed on both ears by an unblinded investigator. Pain was assessed using the Premature Infant Pain Profile. Primary analyses were by intention-to-treat. ClinicalTrials.gov:NCT03650621.

**Findings:** The mean (standard deviation, SD) gestation, birthweight, and postnatal age were (MAA 28(3) vs. placebo 28(2) weeks; MAA 1,057(455) vs. placebo 952(273) g; MAA 7(3) vs. placebo 7(3) weeks. Placebo infants had significantly higher PIPP scores during [mean difference 1.6 points (95%CI 0.1–3.1)] and 1 h mean difference 1.5 points (95%CI 0.7–2.2) after the procedure (*p* < 0.03). Heart rate was lower (173(22) vs. 184(18)/min) and oxygen saturations were higher (93.8(6.2) vs. 91.7(6.1)%, *p* = 0.05) in MAA infants. No adverse effects.

**Interpretation:** MAA may reduce physiological pain responses during and after ROP exam in preterm infants. Assessment of long-term effects are warranted.

**Clinical trial registration**: www.ClinicalTrials.gov, identifier: NCT03650621.

## Introduction

Pain is an inevitable consequence of treatment in a Neonatal Intensive Care Unit (NICU). Critically-ill infants may have to undergo >100 painful and stressful diagnostic procedures within a fortnight and many (~80%) of these are conducted without any or sufficient analgesia (1). Some of these procedures are life-saving and there is no time to provide pre-emptive analgesia. However, others are less critically-needed or indeed, are even planned in advance as part of routine care. Despite this, providing sufficient analgesia is not consistent practice within the NICU and indeed, is often subjective, especially if the infant is young or if the procedure is conducted outside of working hours (2) and without the presence of parents or guardians (2).

This is of great concern because infants who undergo repetitive episodes of untreated or undertreated pain experience adverse outcomes. Pain, of course, causes short-term physiological instability such as tachycardia and respiratory distress which may lead to adverse consequences such as intraventricular hemorrhage (IVH) especially in the sick and fragile preterm infant (3). The long-term impact of unrequited pain is no less debilitating. Children with a history of exposure to high numbers of painful procedures during infancy develop impaired pain processing and poorer neurological and behavioral outcomes despite being otherwise physically healthy (4).

The current analgesic armamentarium within the NICU is limited and is not without complications. Commonly used analgesics such as opioids take time (e.g., up to 10 min) to exert maximum effects and are poorly effective especially in procedures needing rapid onset of analgesia. Many also have serious side-effects including respiratory depression, hypotension (5) and in animal and adult models, neurotoxicity, including increased neuronal death by apoptosis (6). Non-pharmacological strategies such as breast-feeding and swaddling may mitigate the discomfort from less painful procedures but are usually not practical and are usually ineffective for complex and protracted procedures, especially in very sick infants (7).

Seeking alternative and safe forms of analgesia is therefore crucial to ensure optimum short and long-term well-being of sick infants in the NICU. Acupuncture, a field of traditional Chinese medicine (TCM), has been used for thousands of years to provide analgesia for a gamut of illnesses in adults and older children but traditional forms of acupuncture that involve inserting fine needles into specific cutaneous areas is impractical in infants, especially those in the NICU. Acupuncture effects, however, can be implemented by a variety of stimuli, including pressure, heat and magnets and recently, we demonstrated that magnetic auricular acupuncture (MAA), where small magnets are placed around specific points around the ear, significantly decreased procedural pain perception in 40 infants undergoing routine heel pricks. Compared to placebo, MAA was associated with significantly reduced pain scores even after controlling for concomitant analgesia use (e.g., sucrose) (8).

Whether MAA is effective in more protracted and stressful procedures is unknown. In this study, we aimed to examine if MAA compared to placebo administered 1 h before a routine ophthalmological examination for retinopathy of prematurity (ROP) in preterm infants reduced discomfort and pain. We hypothesized that preterm infants receiving MAA compared to placebo would have reduced discomfort or pain during the ROP examination and that MAA, when compared to placebo, would decrease sympathetic stress responses as measured by changes in heart rate and oxygen saturation.

## Methods

### Study Design and Participants

This was a multicentre, blinded, randomized, placebo-controlled trial conducted at three tertiary hospitals in three countries: Royal Hospital for Women, Sydney, New South Wales, Australia, Royal Alexandra Hospital, Edmonton, Canada, and University Malaya Medical Center, Kuala Lumpur, Malaysia. Infants were eligible for inclusion if they were born prematurely before 29 weeks' gestation or had a birth weight below 1,250 g (fulfilling the ROP screening guidelines from each participating hospital), were current inpatients and required ROP screening. Exclusion criteria included cardiorespiratory problems that could impair oxygenation, invasive ventilation, surgery within 14 days prior to ROP examination, major congenital malformations, neurological problems that could impair pain perception, or were treated with opioids or sedatives <24 h before the ROP examination. All infants were assessed for eligibility by the study team. Written informed parental consent was obtained for each infant prior to the study. Approval from each relevant Institutional Review Ethics Boards were obtained from Australia (HREC/18/POWH/442), Canada (Pro00080714), and Malaysia (NMRR-16-143232198).

### Randomization and Masking

We randomized infants to receive either MAA or placebo using a 1:1 randomization, which was stratified by site. A randomization list of unique patient identifiers was generated by the study statistician at Edmonton using a computer-generated random block size. An unblinded study nurse opened a consecutive numbered, sealed, brown envelope containing the unique patient identifier and study group allocation. Researchers, clinicians, outcome assessors, and parents were masked to treatment allocation. In the event of an infant becoming ineligible after randomization, the study was postponed and recommenced when the infant became eligible, without re-randomization.

### Procedure

**Figure 1:**
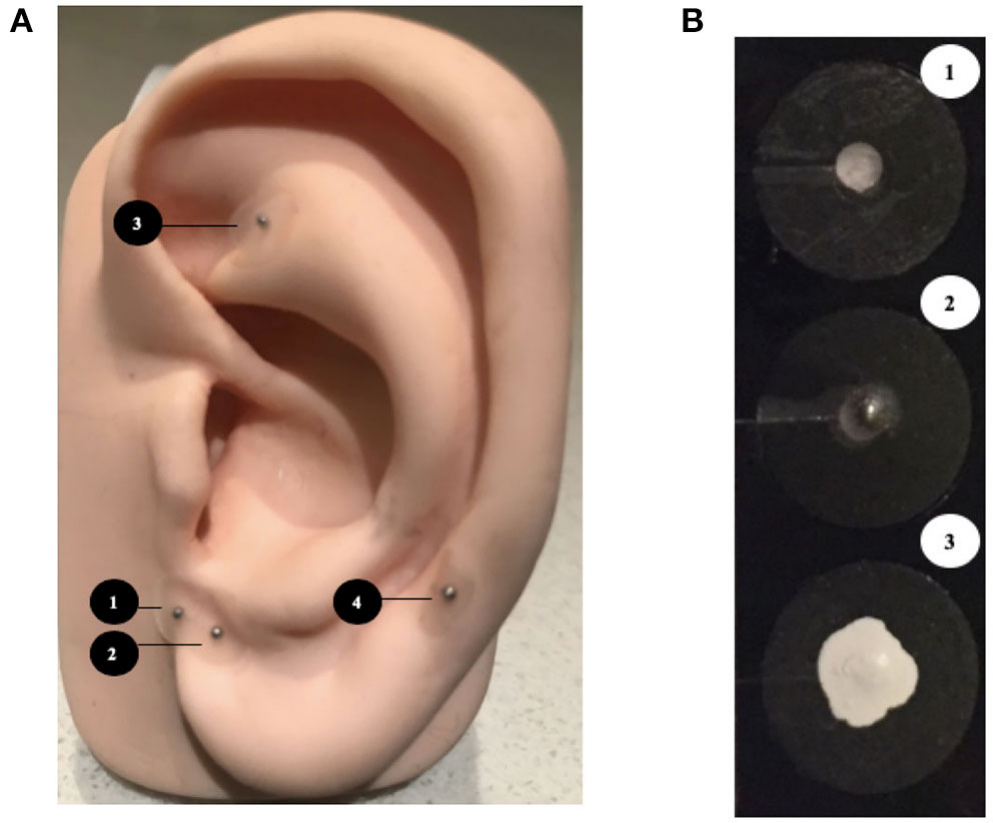
**(A)** Modified Battlefield acupuncture protocol. The magnets were placed sequentially in the following order: (1) Cingulate Gyrus, (2) Thalamus, (3) Shenmen, (4) Cranial Nerve-5 (Ophthalmic branch).9,10 **(B)** (1) The placebo sticker (2) magnet sticker (3) concealment by white correction fluid.

The magnet or placebo stickers were applied to both ears of the infants at least 60 min for the ROP check (Figure 1). In the MAA group, infants had the magnet stickers applied on both ears on four pre-specific auricular acupuncture points, which were modified based on the BFA protocol (9) and included the Cingulate Gyrus (CG) and Thalamus to target central pain modulation, Shenmen for relaxation, and the ophthalmic branch of Central Nerve 5 (10) for sensory innervation of and around the eyes (Figure 1A). Stimulation was performed using Sakamura Magrain Ion Pellets magnets with a strength of 100 Gauss, measuring 1.7 mm in diameter on a 5.0 mm circular sticker (Sakamura, Helio Acupuncture, Japan) (Figure 1B). All personnel applying the magnets were trained in applying the magnets. In the control group, the same stickers were placed at the same pre-specified auricular acupuncture points, but the magnets were removed prior the study. In both groups, the magnet stickers were concealed with white, correction fluid to conceal group allocation (Figure 1B). Each infant was enrolled only once.

The magnet and placebo stickers were removed 60 min after the ROP examination but were replaced by unblinded study nurses if they were displaced before the ROP examination was completed. ROP screening was conducted by the same senior pediatric ophthalmologists at each center. Topical local anesthetic (tropicamide 0.5% and phenylephrine 2.5%) eye drops were instilled 30 min before insertion of an eyelid speculum, and binocular indirect ophthalmoscopic examination was completed using a Flynn style indenter. Infants also received oral sucrose prior ROP screening, and further analgesia was administered based on clinical judgement (i.e., sucrose, swaddling, pacifier).

### Outcomes

The *primary outcome* measure was the difference in pain perception, as assessed by the Premature Infant Pain Profile (PIPP) (11), which was calculated before, during and 60 min after examination of both eyes. A PIPP score of 6 or lower indicates little or no pain and a score >12 indicates moderate to severe pain. Assessment of all determinations of the PIPP score were done by a bedside nurse who was trained in infant pain assessment and who was blinded to group assignment.

*Secondary outcome* measures included changes in heart rate, arterial oxygen saturation, and types of other analgesia administered assess at 60 min prior, during, and 60 min after retinopathy of prematurity screening.

*Safety outcome* measures were adverse effects directly or indirectly related to sticker placement (e.g., skin redness or excoriation, infant irritability, or sticker ingestion). The number of stickers and magnets were counted before removing them after ROP examination.

### Statistical Analyses

Sample size calculation was based on pilot Premature Infant Pain Profile (PIPP) score data that was collected over 3 months in Edmonton with a mean (standard deviation) of 11.7 (3.8). We calculated that a sample size of 74 infants (37 in each arm) with 80% power (*p* < 0.05; two-tailed) would allow for detection of 20% reduction in pain scores. To account for non-normality of the data the sample size was increased to 90 infants (45 in each arm). The sample size was inflated by 10% to 100 (50 in each arm) to account for 10% dropout.

Trial outcomes were analyzed and reported according to the trial protocol and statistical analysis plan (version 2.0). Analysis was intention-to-treat principle for all our analyses, and a *p*-value of 0·05 (two-sided 5% significance level) was deemed significant for all outcome measures. We report mean (SD) or median (IQR) according to whether data were normally distributed or skewed, and relative and absolute frequencies are used for categorical variables. Linear models were used for the analysis of the primary outcome with baseline pain score included as a covariate. Linear mixed models (SAS procedure MIXED) were used to examine the effect of the randomization group on all determinations of the PIPP pain score both during and after the ROP exam. To account for clustering of patients within centers, sites were entered as a random effect. Models were chosen for the smallest Akaike Information Criterion (AIC). In all models, residuals were approximately normally distributed. All statistical analyses were performed by SAS 9.4 (SAS Institute Inc., Cary, NC, USA). The trial was registered at ClinicalTrials.gov (NCT03650621).

### Role of the Funding Source

The funder had no role in study design, data collection, data analysis, data interpretation, or writing of the report. The corresponding author had full access to all data in the study and had final responsibility for the decision to submit for publication.

## Results

**Figure 2:**
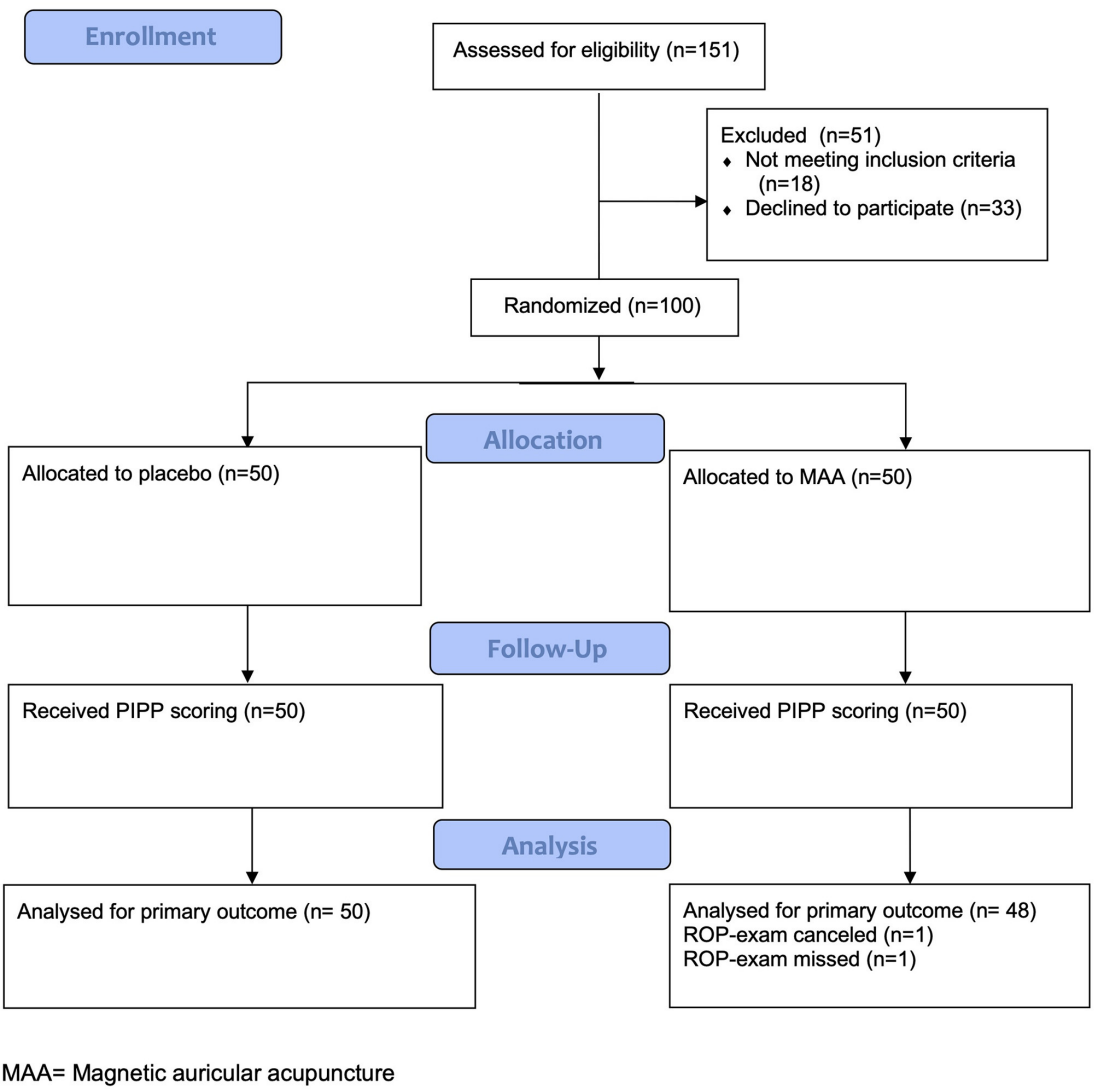
Consort flow diagram.

Between August 29, 2018 to July 30, 2019, a total of 151 patients were screened. Reasons for exclusion are provided in the consort diagram (Figure 2). A total of 100 infants were randomized to either MAA (*n* = 50) or placebo (*n* = 50). After randomization, two infants were excluded (*n* = 1 ROP-exam canceled, *n* = 1 ROP-exam missed), which left 98 infants for analysis on an intention-to-treat for the primary outcome (MAA: 48, Placebo: 50). Infant demographics were well-balanced and are reported in Table 1.

**Table 1:** Baseline characteristics of the intention-to-treat population.

	**MAA (*n =* 48)**	**Placebo (*n =* 50)**	***p*-value**
**Infant**			
Male	40 (63%)	32 (47%)	0.08
GA (week)	27.8 (2.7)	27.2 (2.2)	0.18
Corrected age at examination (week)	34.9 (2.0)	34.6 (2.3)	0.27
Postnatal age (week)	7.1 (3.2)	7.4 (3.2)	0.50
Birth weight (g)	1,014 (296)	952 (273)	0.21
5 min Apgar Score	7.4 (2.1)	7.7 (1.7)	0.50
**Maternal**			
Age (years)	32.4 (5.7)	31.6 (5.4)	0.40
Mean parity	1.9 (1.7)	1.7 (1.6)	0.31
Cesarean Section	44 (69%)	38 (56%)	0.13

*Data are expressed as mean (SD) unless indicated or n (%). MAA, Magnetic auricular acupuncture; GA, gestational age*.

Compared to MAA infants, those in the placebo group had significantly higher PIPP scores during (mean difference 1·6 points (95% CI 0.1–3.1) and 1 h (mean difference 1.5 points (95% CI 0.7–2.2), *p* = 0.03 after the ROP examination (Table 2). The mean (SD) PIPP score pre-examination was 1.3 (1.5) and 1.3 (1.8) in the control and intervention group, respectively. During ROP-examination the PIPP score were 13.5 (3.7) and 11.9 (4) and 1 h afterwards PIPP scores were 3.1 (2.2) and 1.6 (1.6) in the control and intervention group, respectively.

**Table 2:** Difference in PIPP scores during the eye exam.

**Variable**	**Estimate**	**95% CI for the estimate**		***p*-value**
		**Lower**	**Upper**	
Intercept	10.9	9.7	12.1	<0.0001
Pain baseline score	0.7	0.3	1.2	0.002
Group (Placebo vs. MAA)	1.6	0.1	3.1	0.03

*MAA, magnetic auricular acupuncture; Cl, confidence interval*.

*Linear mixed models with random effects*.

Mean (SD) heart rate was significantly lower in the MAA group compared to the placebo group during the examination [172.7 (21.6) vs. 184.3 (17.6) beats/min *p* < 0.001, respectively]. Similar, oxygen saturation was significantly higher in the MAA group compared to control group 93.8 (6.2) vs. 91.7 (6.1), *p* = 0.05, respectively. There was no difference in the use of additional analgesia (Table 3).

**Table 3:** Secondary outcomes.

**Variables**	**MAA (*n =* 50)**	**Placebo (*n =* 48)**	***p*-value**
**Heart rate (bpm)**			
Before	148.8 (13.7)	150.0 (16.0)	0.65
During	172.7 (21.6)	184.3 (17.6)	<0.001
After	148.2 (15.9)	153.0 (12.1)	0.06
**Oxygen saturation (%)**			
Before	96.8 (2.9)	96.87 (2.9)	0.84
During	93.8 (6.2)	91.71 (6.1)	0.05
After	96.6 (3.1)	95.78 (5.0)	0.25
**Analgesia**			
Oral sucrose	11 (17.2%)	15 (22.1%)	0.48
Pacifier	20 (31.3%)	18 (26.5%)	0.54
Breastmilk	2 (3.1%)	5 (7.4%)	0.30
Swaddling	16 (25.0%)	17 (25%)	1.00

*MAA, magnetic auricular acupuncture. Data are expressed as mean (SD) or n (%). Analgesia n (%) represents analgesia given immediately before ROP examination*.

We assessed adverse effects directly or indirectly related to sticker placement in all patients who received the stickers. One infant in the placebo group experienced skin redness on the ear and one infant had one sticker dislodged. We did not observe irritability, skin excoriation or inadvertent ingestion of magnets.

## Discussion

Our study showed that MAA significantly reduced scores associated with pain, discomfort and distress in preterm infants during and up to 1 h after the ROP examination. In addition, the application of MAA resulted in improved sympathetic pain response as indicated by lower heart rates and higher oxygen saturations when compared to placebo. We previously showed that MAA, when applied according to the BFA protocol, reduced PIPP scores in premature infants undergoing heel pricks (8). Heel pricks, however, are rapid procedures, usually completed in <30 s. Whether MAA is efficacious for more protracted and stressful procedures in the premature infant population is uncertain. In this study, we examined the use of MAA for the ROP examination, which is considered one of the most stressful procedures conducted on relatively healthy preterm infants. To date, no intervention has been shown to be effective in alleviating infant distress during or after the ROP examination (12). Indeed, a study in which infants were randomized to either morphine (*n* = 15) or placebo (*n* = 16) as analgesia for ROP examination was ceased due to morphine-associated respiratory depression with no additional analgesic efficacy (13).

The use of acupuncture within the NICU is not new practice. Various modalities for different procedures (14) have been examined by previous studies but definitive evidence for the efficacy, applicability, and acceptability of acupuncture has been impeded by study heterogeneity (15–17). In addition, there is also little information about the impact of acupuncture on longer term outcomes, including neurodevelopment and future studies should take these knowledge gaps into account. In this study, we chose magnets as the acupuncture vehicle because the stickers were cheap, easily placed by trained personnel and did not interfere with routine newborn care, unlike other acupuncture modalities like needles. Whether other forms of acupuncture e.g., needles, laser, heat would have been similarly or more effective is uncertain and needs further study.

Despite these uncertainties, the need to develop safe and efficacious analgesic treatments in the NICU is paramount. Infants requiring intensive care are usually subject to multiple episodes of pain, where analgesia is provided on *ad-hoc* and subjective basis (1, 2). Clinicians rely predominantly on pharmacological forms of analgesia that are neurotoxic even to the mature adult brain (6). Despite the side effects, medications like morphine may not be effective even for innocuous and commonplace procedures such as heel pricks (18) for a variety of reasons: inconvenience (e.g., needing intravenous access), prolonged lag-time for peak effect and concomitant side-effects. Stress caused by less serious procedures may be alleviated by non-pharmacological methods such as breast-feeding, swaddling and sucrose but these strategies are ineffective with more serious pain, for unexpected procedures especially if the infant is critically unwell (7, 19).

In MAA, the magnets can be left on the infant for days to pre-empt pain. In Chen's study, magnets were left on the ear for 3 days because each infant could have multiple heel pricks and placing stickers each time an infant had a heel prick was impractical (8). In the current study, MAA stickers were only left on for 1 h as the infant was unlikely to have an ROP examination for at least another 1–2 weeks. Acupuncture effects are augmented with prolonged or repeated application by increasing resilience and consolidating central pain control (19, 20).

The site of magnet placement could also influence pain reaction. Chen et al. (8) used the BFA protocol, which has been shown to relieve even serious pain, e.g., war wounds in adults (20). In this study, we used four points known to influence central pain perception (i.e., brainstem nuclei, thalamus, cingulate gyrus, and somatosensory cortex) through stimulation of auricular branches of cranial nerves 5 and 10 (21–23). Whether other protocols are similarly effective need to be elucidated, especially for the involvement of cranial nerve 10, which plays a critical role in parasympathetic cardiac control that influences heart rate or desaturations during pain stimuli (24). Auricular stimulation of fibers of the cranial nerve 10 might also modulate cardiovascular function and potentially improve physiological deterioration during painful procedures (25).

Our study only used one pain assessment tool, which is a limitation of the study. Some aspects of the score are influenced by behavioral components and may be subjective depending on assessor objectivity (26). Nevertheless, the analgesic effect of MAA was apparent even in non-subjective parameters like heart rates and arterial oxygen saturations.

Applied pressure from stickers exerts only very minimal surface stimulation. The combined pressure from the sticker and magnet is unlikely to be much more than pressure from the sticker alone and therefore, we postulate that magnetic stimulation was responsible for the observed effect. (27) reported that in the adult mouse model using dorsal root ganglion neuronal cell culture, exposure to a static magnetic field inhibits elicited action potentials, which might explain the potential effect of magnetic field on pain perception (28). We also used the weakest (100G) magnets available to minimize the risk of theoretical side-effects, including discomfort and skin irritation but much stronger magnets have been used in term infants and adults without complications (29).

Our study has limitations. There are obviously a number of variations in how the magnets can be used for analgesia in newborn infants, including duration and protocol of magnet application. Magnet strength was also a potential confounder. Our results are generalizable only to the infants that met our study inclusion criteria and do not account for the effects of MAA with repeated treatment as this can increase resilience and consolidate pain control.

In conclusion, MAA is non-invasive, affordable, portable, can easily be administered in a busy NICU setting. It is also ideal in situations requiring rapid delivery of effective non-pharmacological analgesia (22). Education and training in the use of MAA can readily be provided to clinical staff by certified acupuncturists within a day but potential barriers regarding its uptake, including staff and consumer education and ideation, need to be addressed prior to its wider-spread implementation. Further studies are needed to determine the effects of MAA and other forms of acupuncture on different stressful and painful procedures and on long-term neurodevelopmental and behavioral outcomes.

## Data Availability Statement

The original contributions presented in the study are included in the article/Supplementary Materials, further inquiries can be directed to the corresponding author/s.

## Ethics Statement

The studies involving human participants were reviewed and approved by Approval from each relevant Institutional Review Ethics Boards were obtained from Australia (HREC/18/POWH/442), Canada (Pro00080714), and Malaysia (NMRR-16-143232198). Written informed consent to participate in this study was provided by the participants' legal guardian/next of kin.

## Author Contributions

KG: ethics approval, patient-recruitment, performed randomization and allocation concealment, administered intervention, performed statistical analysis, interpretation of results, drafted initial report, critical revision, and approval of final report. J-LO: project idea, ethics approval, analysis, interpretation of results, critical revision, and approval of final report. IQ-S: project idea and acupuncture protocol, interpretation of results, critical revision, and approval of final report. AK: project idea, interpretation of results, critical revision, and approval of final report. AL: project idea, patient-recruitment, performed randomization and allocation concealment, analysis, interpretation of results, critical revision, and approval of final report. KDL: project idea, ethics approval, interpretation of results, critical revision, and approval of final report. KL: patient-recruitment, collecting of outcome data, critical revision, and approval of final report. MD and NG: patient-recruitment, collecting of outcome data, interpretation of results, critical revision, and approval of final report. AM: project idea, collection and assembly of data, interpretation of results, critical revision, and approval of final report. MY: project idea, analysis of data, interpretation of results, critical revision, and approval of final report. GS: project idea, ethics approval, collection and assembly of data, interpretation of results, critical revision, and approval of final report. All authors contributed to the article and approved the submitted version.

## Conflict of Interest

The authors declare that the research was conducted in the absence of any commercial or financial relationships that could be construed as a potential conflict of interest.

## Abbreviations

ROP: Retinopathy of prematurity
AA: Auricular acupuncture
BFA: Battlefield Acupuncture
MAA: Magnetic auricular acupuncture
PIPP: Premature Infant Pain Profile
N-PASS: Neonatal Pain Agitation and Sedation Scale
SD: Standard Deviation
CI: Confidence Interval.
